# Fetal imaging approach to spinal dysraphism diagnosis

**DOI:** 10.1007/s00247-025-06287-2

**Published:** 2025-06-20

**Authors:** Usha D. Nagaraj, Karin S. Bierbrauer, Beth M. Kline-Fath

**Affiliations:** 1 Clinical Radiology Department of Radiology and Medical Imaging, Cincinnati Children’s Hospital Medical Center, 3333 Burnet Avenue, Cincinnati, OH 45229-3026 USA; 2 University of Cincinnati Medical Center, Cincinnati, USA

**Keywords:** Fetal, Magnetic resonance imaging, Neural tube defects, Spinal dysraphism

## Abstract

Prenatal spinal dysraphism is a common reason for referral for fetal magnetic resonance imaging (MRI). Fetal MRI can not only help confirm the diagnosis but also aid in classification and identification of imaging findings that help guide prenatal counseling and perinatal management. Open spinal dysraphic defects, or those that are not skin-covered, are potential candidates for prenatal surgery. Closed, or skin-covered, spinal dysraphic defects occur in many forms and can be classified by the presence or absence of a sac on fetal imaging. Closed spinal dysraphism identified without a sac on fetal imaging is typically complex in nature, with simple forms being less commonly diagnosed in the prenatal period. In this article, we will review a method of approaching the classification of fetal spinal dysraphism with a focus on MRI findings and review examples of some of the most common entities encountered in clinical practice.

**Graphical Abstract:**

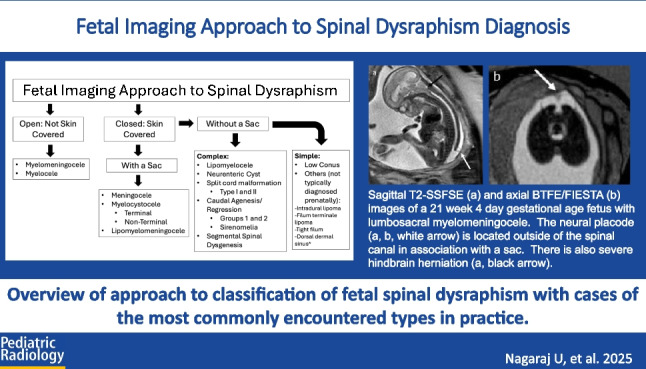

**Supplementary Information:**

The online version contains supplementary material available at 10.1007/s00247-025-06287-2.

## Introduction

Congenital abnormalities of the spine are one of the most common reasons for referral for fetal MRI and comprise one of the most common fetal anomalies of the central nervous system identified [1]. Congenital spine anomalies go by a variety of different names, including neural tube defects, spina bifida, and spinal dysraphism. There are many different forms of spinal dysraphism described, which vary widely in imaging appearance and clinical presentation, making accurate imaging descriptions of great importance. The clinical-neuroradiological classification of spinal dysraphism proposed by Tortori-Donati et al. in 2000 remains the most widely cited in the radiology literature, dividing spinal dysraphism into those defects that are open (not skin-covered) and closed (skin-covered), then subdividing the skin-covered spinal dysraphic defects into those with and without a subcutaneous mass [2–4]. While this system has allowed for relatively consistent nomenclature to help facilitate accurate diagnosis, its design is based on postnatal imaging features combined with postnatal clinical features, making it challenging to incorporate into a fetal imaging template. This review article will aim to illustrate a proposed method of approaching spinal dysraphism on fetal imaging with a focus on MRI findings and review some of the most common types of fetal spinal dysraphism encountered in clinical practice. While the included examples will primarily be fetal MRI images, additional ultrasound images for the included cases, including video clips, can be found in the Supplementary Materials.

## Imaging modalities

Ultrasound is the mainstay of imaging diagnosis in the fetus and can provide important information about spinal dysraphism, including the presence and size of an associated sac, degree of skin covering of the defect, and the osseous spinal level of the defect [5]. Though fetal ultrasound serves an important role in screening, fetal MRI is the preferred imaging technique, allowing for a more comprehensive assessment of fetal spinal dysraphism. Fetal MRI provides a better assessment of the brain and spinal cord than ultrasound and is not limited by maternal body habitus, amniotic fluid volume, and fetal positioning to the same degree as ultrasound [6]. Recommended standard imaging protocols for fetal spine MRI have been described in the literature [7, 8]. In our practice, the fetal spinal dysraphism protocol includes T2 single-shot fast spin echo (SSFSE) and 2D balanced turbo-field-echo/fast imaging employing steady-state acquisition (BTFE/FIESTA) images of the spine with 3–4-mm-thick slices in the axial, sagittal, and coronal planes, with the sagittal and axial BTFE/FIESTA images repeated twice or more to the radiologists’ satisfaction. Our imaging protocol also includes echo planar imaging (EPI) black bone images of the spine and T1-weighted images of the body in the sagittal and coronal planes (Supplementary Table S1) [9].

While MRI is the preferred imaging technique for the fetal central nervous system (CNS), ultrasound (US) and MRI go hand in hand offering complementary information and are best utilized together. Fetal US offers standardized assessments of amniotic fluid volume, fetal growth parameters, color and spectral Doppler evaluation, fetal heart rate and biophysical profile, and maternal cervical length, all of which contribute to the overall assessment of fetal well-being and clinical management [10, 11]. One example of how fetal MRI and US can be used together is in the assessment of anorectal malformation. T1-weighted images are particularly helpful on MRI in assessing for co-existing anorectal malformations, which are common in patients with spinal dysraphism [12]. The presence of a normal column of rectal meconium signal below the urinary bladder can help exclude anorectal malformation [13]. In conjunction, the presence of a normal anal dimple on fetal ultrasound can also help exclude anorectal malformation [14].

## Fetal imaging approach to spinal dysraphism diagnosis

Much like the clinical-neuroradiological classification scheme, one of the most important goals of the fetal imager is to determine if a spinal dysraphism is open (not-skin-covered) or closed (skin-covered). However, unlike the clinical-neuroradiological classification, the documented physical exam findings of an open defect (exposed neural tissue with leaking cerebrospinal fluid (CSF)) or closed defect (skin-covered) are not available in the fetus. Prenatal distinction, however, is of particular importance when determining candidacy for prenatal repair, as this technique is currently not offered to patients with closed spinal dysraphism [15]. Details regarding fetal imaging findings in relation to fetal surgery are not discussed in this review; however, they have been previously described by the authors in other reviews [16, 17].

Historically, elevated maternal serum alpha-fetoprotein (AFP) has been a helpful screening tool in diagnosing fetal open spinal dysraphism (OSD), though it is used less frequently in recent years due to the increased use of non-invasive prenatal testing (NIPT) [18]. The diagnosis of OSD is usually highly accurate by demonstrating secondary brain findings on fetal ultrasound. Chiari II malformation, by identification of the “lemon sign,” or bifrontal concavity of the calvarium, is the most sensitive and is seen in up to 98% of patients with OSD before 24 weeks gestational age [19]. The “banana sign,” the description given to the appearance of the cerebellum wrapping around the brainstem, is another sensitive sign for detecting OSD prenatally [19]. Although most spinal dysraphic defects can usually be identified on prenatal ultrasound, it can be difficult to determine if the neural tube defect is open or closed. Fetal MRI is helpful as OSD tend to have a thinner sac wall, lack of sac wall continuity with the adjacent epidermal and subcutaneous tissues, a typical imaging appearance of the cord in the defect, and more hindbrain herniation below the foramen magnum [20]. Amniotic fluid studies looking at elevated levels of AFP, expressed as multiples of the median (MoM), and the presence of acetylcholinesterase (AChE) can be helpful in cases of uncertainty, especially in patients who are being evaluated for candidacy for prenatal surgery [21].

**Fig. 1:**
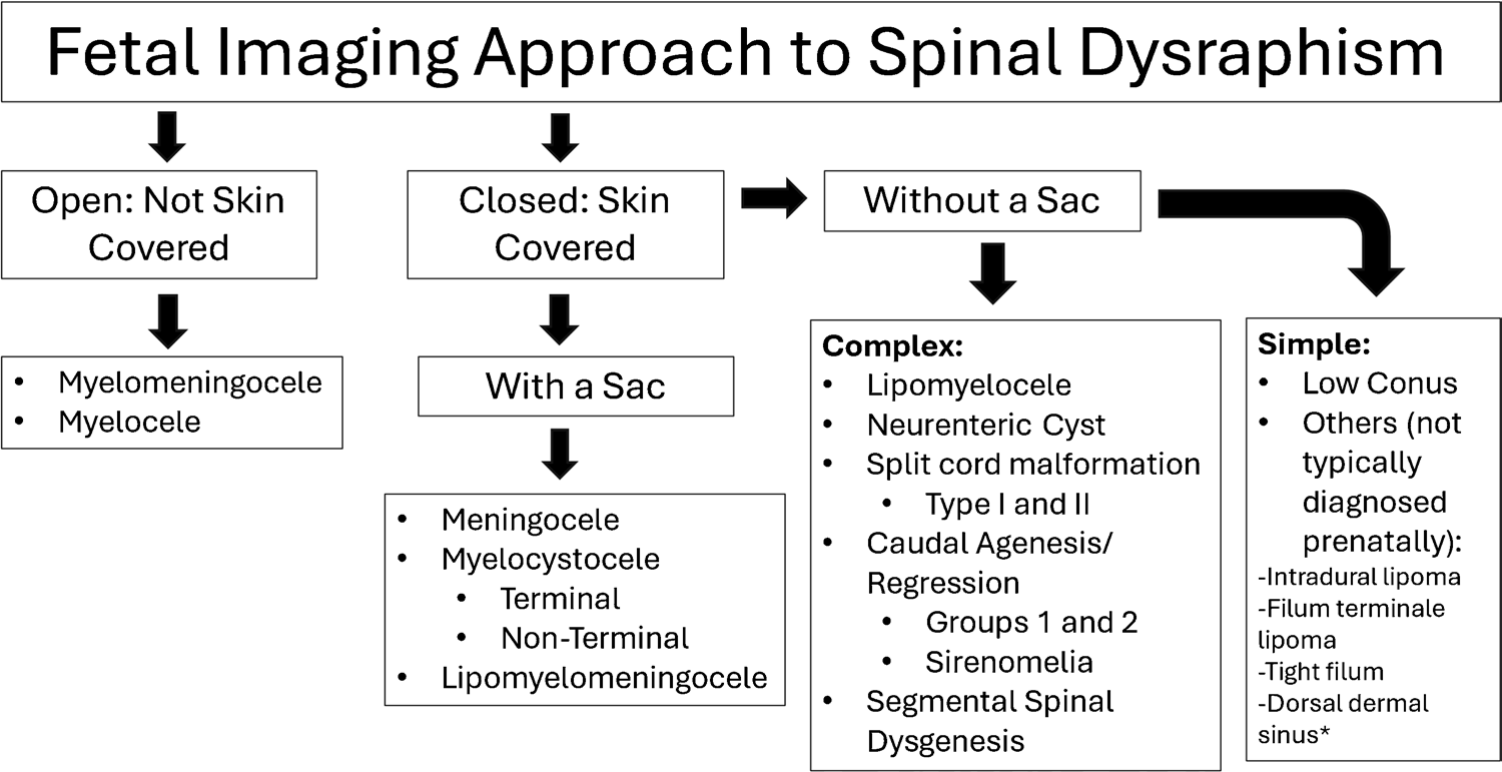
Overview of approach to classification of fetal spinal dysraphism. This is listed as “complex” in the Tortori-Donati et al. clinical-neuroradiological classification, though listed as “simple” in a subsequent review by Rufener et al. [2, 4] (*asterisk*)

In the clinical-neuroradiological classification described by Tortori-Donati et al., closed spinal dysraphism (CSD) was subdivided based on the presence or absence of a subcutaneous mass, presumably based on physical exam findings. In the fetus, physical examination is not feasible, and the presence or absence of a subcutaneous mass may be unclear by imaging due to the relative underdevelopment of fetal fat, particularly in the second trimester [22]. A fluid-filled sac, on the other hand, can be more reliably identified on fetal imaging than a subcutaneous mass and can serve as a marker to help in CSD classification. In the fetus, one can subdivide CSD into those with a sac containing at least some visible CSF (meningocele, lipomyelomeningocele, myelocystocele) and those without a sac. CSD without a sac may then be further categorized in a fashion similar to the clinical-neuroradiological classification as “complex” or “simple.” Complex CSD are those with associated abnormalities outside of the spinal canal and include lipomyelocele, neurenteric cyst, split cord malformation, caudal agenesis, and segmental spinal dysgenesis. Simple CSD, such as an isolated tight of fatty filum, is unlikely to be diagnosed prenatally in isolation, though may present with a low conus on fetal MRI (Fig. 1, Table 1).

**Table 1:** Description of spinal dysraphism seen on fetal imaging

Spinal dysraphism	Description
OSD: myelomeningocele	• OSD with sac • Neural placode outside spinal canal
OSD: myelocele	• OSD without a sac • Neural placode flush with skin surface or within spinal canal
CSD with sac: meningocele	• CSD with CSF-filled sac not containing any neural elements • Conus may be normal or low-lying
CSD with sac: myelocystocele	• Terminal and non-terminal subtypes • Terminal myelocystocele: -End of spinal cord splits to form a fluid collection which connects with central canal of spinal cord (a.k.a. “cyst within a cyst”) -Associated with OEIS • Non-terminal myelocystocele (2 subtypes): -Abortive form: sac traversed by a fibroneurovascular stalk between spinal cord and dome of sac -Complete form: hydromyelic cavity in sac continuous with spinal cord syrinx
CSD with sac: lipomyelomeningocele	• CSD with spinal cord elongation terminating in placode-lipoma interface • Placode lipoma interface outside spinal canal with expansion of surrounding meninges creating sac
CSD without sac complex: lipomyelocele	• CSD with spinal cord elongation terminating in placode-lipoma interface • Placode-lipoma interface inside spinal canal, no fluid-filled sac
CSD without sac complex: neurenteric cyst	• Paraspinal or intraspinal cyst lined by endoderm derivatives • Associated with segmentation anomalies
CSD without sac complex: split cord malformation	• Sagittal division of spinal cord, 2 types • Type I: −2 separate dural tubes -Usually with bony or cartilaginous cleft • Type II: -Single dural tube -Usually with a thin separating fibrous band
CSD without sac complex: caudal agenesis/regression	• Absence of caudal spine, 2 groups • Group 1: -High blunted conus -More severe associated anomalies (osseous, GI, GU) • Group 2: -Low-lying tethered cord
CSD without sac complex: segmental spinal dysgenesis	• Focal kyphosis or disarticulation of spine • Thin or absent spinal cord at defect level • Thick cord caudal to defect
CSD without sac simple: low conus	• Lower than L4 in 2nd trimester, L3 in 3rd trimester • Conus lower than inferior renal poles

*OSD* open spinal dysraphism, *CSD* closed spinal dysraphism, *OEIS* omphalocele-exstrophy-imperforate anus-spinal defects, *GI* gastrointestinal, *GU* genitourinary

## Open spinal dysraphism

**Fig. 2:**
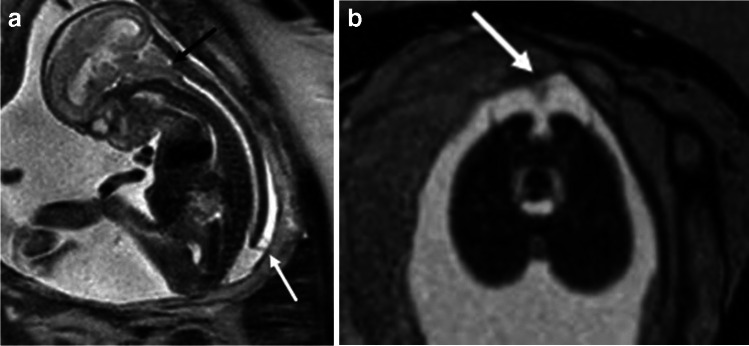
**a**-**b** Fetal MRI of myelomeningocele. Sagittal T2-SSFSE (**a**) and axial BTFE/FIESTA (**b**) images of a 21-week 4-day gestational age fetus with lumbosacral myelomeningocele. The neural placode (**a**, **b**, *white arrow*) is located outside of the spinal canal in association with a sac. There is also severe hindbrain herniation (**a**, *black arrow*)

**Fig. 3:**
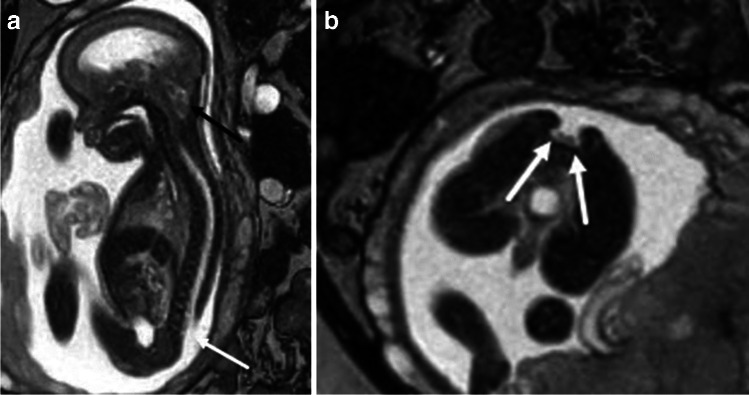
**a**-**b** Fetal MRI of myelocele. Sagittal (**a**) and axial (**b**) BTFE/FIESTA images of a 22-week 3-day gestational age fetus with lumbosacral myelocele marked absence of a sac (**a**, *white arrow*). The neural placode is within the spinal canal with co-existing type II split cord malformation (**b**, *white arrows*). There is also severe hindbrain herniation (**a**, *black arrow*)

OSD is a congenital abnormality of the spine that is not covered by skin, which results in abnormal exposure of the neural placode (incompletely neurulated portion of the spinal cord) and its associated nerve roots to the amniotic fluid in utero. As a result, CSF from the spinal canal leaks through the defect in utero, causing Chiari II malformation, which is a constellation of imaging intracranial findings including hindbrain herniation [23]. OSD may be partially covered by skin, and in certain cases may be difficult to differentiate on neonatal exam from CSD that ruptured prenatally or during the birthing process. Though OSD as a whole are most commonly referred to as “myelomeningoceles” in the literature, myelomeningocele and myelocele are the two main categories of OSD described in the clinical-neuroradiological classification [2]. In myelomeningoceles, the neural placode is outside the spinal canal in association with a thin-walled sac (Fig. 2). In myeloceles, also known as myeloschisis, the neural placode is either within the spinal canal or flush with the skin surface (Fig. 3). This distinction has surgical implications, as prenatal skin closure tends to be technically more feasible with myelomeningoceles because of the excess tissue associated with the placode, decreasing the need for graft closure when compared to myeloceles [24]. On fetal ultrasound, more clubfoot deformity has been observed with myelomeningoceles when compared to myeloceles [25]. On fetal MRI, more hindbrain herniation is observed with myeloceles when compared to myelomeningoceles [26]. However, the long-term clinical implications of these fetal imaging findings are not yet known.

While fetal ultrasound can usually identify the myelomeningocele sac in OSD, myeloceles can be more challenging to see, particularly in small sacral defects. Fetal MRI often offers excellent delineation of the neural placode along with an assessment of the remaining spinal cord anatomy, including the presence of syrinx or co-existing split cord malformation [27]. The myelomeningocele sac wall tends to be thin and sometimes nearly imperceptible on fetal MRI. Myelomeningocele is usually associated with hindbrain herniation, though it is important to know that 8% of fetuses with OSD do not have hindbrain herniation [20, 26]. The vast majority of OSD are lumbosacral in location, though some can be non-terminal or segmental in location. This appearance can be best explained by a multisite closure theory of neural tube formation [28].

Identifying the upper level of the osseous defect of OSD is important for prenatal counseling and determining the best candidates for fetal surgery, since higher and larger defects correlate with increased risk of lack of childhood ambulation [29]. Many fetal imagers feel most comfortable with ultrasound utilization for level assignment due to the increased echogenicity of osseous structures and ultrasound’s ability to consistently identify the T12 landmark as the lowest rib-bearing thoracic vertebral body. 3D ultrasound can also be of potential benefit over 2D ultrasound in identifying the exact level of the osseous spinal dysraphic defect, allowing a single volume acquisition to be viewed in the axial, sagittal, and coronal planes simultaneously [30]. 3D ultrasound also has the added benefit of providing patients with more identifiable images and keepsake memories, allowing for improved patient satisfaction [31]. Despite these potential benefits, in published literature, fetal MRI has been shown to be just as accurate in level assignment as fetal ultrasound [27, 32]. In addition to anatomic defect level, the pattern of lower extremity movement on ultrasound has the potential to be a predictor of future independent ambulation, especially with prenatal intervention. Examination for absence or presence of ankle plantar flexion (S1), ankle dorsal flexion (L5), knee flexion (L4), knee extension (L3), hip adduction (L2), and hip flexion (L1) for prenatal functional level assignment has been described and can be implemented clinically by documenting the lowest level of function [33].

## Closed spinal dysraphism: with a sac

### *Meningocele*

**Fig. 4:**
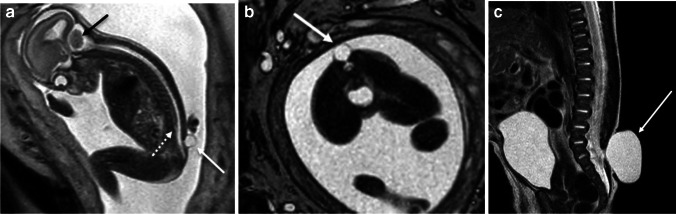
**a**-**c** Fetal MRI of meningocele. Sagittal T2-SSFSE (**a**) and axial BTFE/FIESTA (**b**) images of a 22-week gestational age fetus with a sacral meningocele (*white arrow*). There are normal positioning of the conus medullaris (**a**, *white dashed arrow*) and no hindbrain herniation (**a**, *black arrow*) or other features of Chiari II malformation. Sagittal T2-TSE image of the same patient at 1 day of age confirming the sacral meningocele (**c**, *white arrow*) which was skin covered on physical exam

This CSD is characterized by a skin-covered CSF-filled sac lined by dural and arachnoid mater in continuity with the spinal canal. The meningocele sac should classically not contain any neural elements, though nerve roots may potentially be displaced into the meningocele sac [34]. Meningoceles can be located dorsal, lateral, or anterior to the spinal canal. Dorsal meningoceles can be difficult to differentiate from myelomeningoceles because they can have a relatively thin sac wall dome on fetal imaging; however, unlike OSD, they have a normal appearing spinal cord and are not associated with hindbrain herniation or other features of Chiari II (Fig. 4). The continuous sac-wall sign, seen in closed spinal dysraphism marked by gradual smooth tapering transition between the sac wall and adjacent subcutaneous fat and epidermal soft tissues, may potentially be helpful in differentiating this entity from an OSD. Imaging schematic for the continuous sac-wall sign can be found in a publication by Nagaraj et al. in 2016 [20]. Anterior meningoceles are rare, though they can occur anywhere along the spine, most commonly anterior to the sacrum in the fetus [35]. These can be seen in association with sacral dysgenesis (caudal agenesis) and anorectal malformation, referred to as the Currarino triad [36, 37]. T1-weighted images can be useful in the assessment of co-existing anorectal malformations in these patients.

### Terminal myelocystocele

**Fig. 5:**
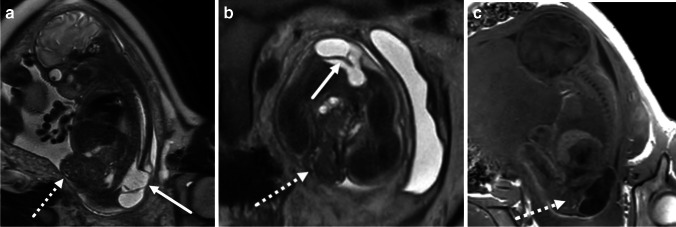
**a**-**c** Fetal MRI of terminal myelocystocele in omphalocele exstrophy imperforate anus spinal defect (OEIS) complex. Sagittal (**a**) and axial (**b**) T2-SSFSE images in a 31-week 5-day gestational age fetus with a lumbosacral terminal myelocystocele (**a**, **b**, *white arrow*) with the sac demonstrating a “cyst within a cyst” appearance and the placode demonstrating a “trumpet bell” morphology (**b**). There are sacral hypoplasia (**a**) and absence of the urinary bladder with a lower anterior abdominal wall defect (**a**, **b**, *dashed arrow*) consistent with bladder exstrophy. Sagittal T1-weighted image (**c**) demonstrates absence of normal T1 hyperintense rectal meconium signal (*dashed arrow*) consistent with co-existing anorectal malformation

Terminal myelocystoceles are a CSD involving the lumbosacral spinal cord in which the skin-covered defect contains herniated neural placode with syringohydromyelia creating a “cyst within a cyst” appearance. While these can be difficult to differentiate from lipomyelomeningoceles on fetal MRI, one key difference is that in terminal myelocystoceles the neural placode splits creating a “trumpet bell” morphology (Fig. 5b) [38]. Unlike other CSD, terminal myelocystoceles have a much higher incidence of hindbrain herniation with an incidence as high as 40% [39, 40]. Also, terminal myelocystoceles have an increased association with caudal agenesis spectrum and cloacal malformations (omphalocele-exstrophy-imperforate anus-spinal defects or OEIS), which can also help in diagnosis (Fig. 5) [20]. The OEIS complex is a rare congenital malformation for which urgent abdominopelvic repair in the immediate neonatal period can be life-saving. Terminal myelocystoceles are one of the most common CSD in association with OEIS and, unlike other CSD, require early surgical intervention once recuperation from abdominopelvic surgery has been achieved [41]. This is because there is a known risk of early rapid loss of lower extremity motor function and increasing sac size with terminal myelocystoceles, making early diagnosis important [42].

### Non-terminal myelocystocele

**Fig. 6:**
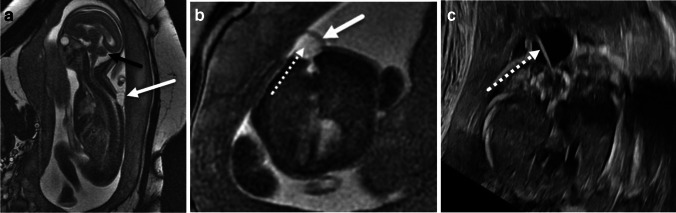
**a**-**c** Fetal MRI and US of non-terminal myelocystocele, abortive subtype. Sagittal (**a**) and axial (**b**) BTFE/FIESTA images of a 22-week 2-day gestational age fetus with mid thoracic non-terminal myelocystocele. There is no hindbrain herniation (**a**, *black arrow*) or other features of Chiari II malformation, and there is faint visualization of a fibroneural stalk in the sac (**b**, *dashed arrow*) with normal appearance of the spinal cord. Axial image from the corresponding fetal ultrasound better delineates the fibroneural stalk (**c**, *dashed arrow*) confirming the diagnosis

**Fig. 7:**
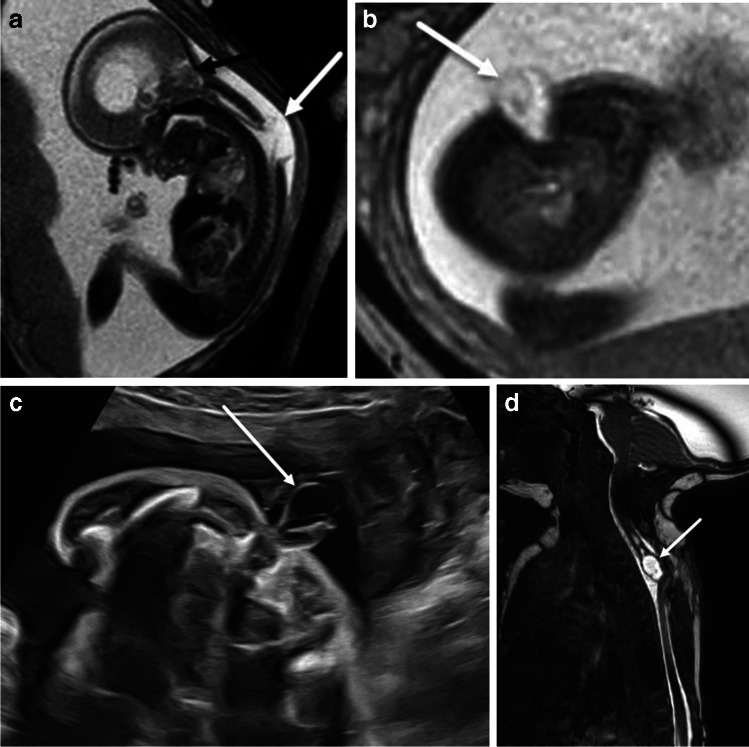
**a-d** Fetal MRI and US of non-terminal myelocystocele, complete form. Sagittal (**a**) and axial (**b**) BTFE/FIESTA images of a 22-week 3-day gestational age fetus with an upper thoracic non-terminal myelocystocele (*white arrow*) with a “cyst within a cyst” appearance consistent with herniation of hydromyelic cavity. There is severe hindbrain herniation noted (**a**, *black arrow*). Axial image from the corresponding fetal ultrasound again shows the “cyst within a cyst” appearance of the thoracic myelocystocele (**c**, *white arrow*). Sagittal BTFE/FIESTA image (**d**) of the same patient at 12 days of age status post repair showing the associated syrinx (*white arrow*) and persistent hindbrain herniation

Non-terminal myelocystoceles are a CSD that occur at locations other than the end of the spinal cord and are focal, skin-covered defects containing CSF and neural elements [43]. These are focal defects primarily affecting the cervical, thoracic, or upper lumbar spine. Rossi et al. described two subtypes of non-terminal myelocystoceles: one is the abortive form or “myelocystocele manqué” in which a skin-covered CSF-filled sac is crossed by a fibroneurovascular stalk that extends from the dorsal aspect of the spinal cord to attach to the dome of the sac (Fig. 6). This entity has also been described under the category “limited dorsal myeloschisis” by Pang et al., who describe a heterogeneous group of CSD under this category, though this term is not included in the above-referenced clinical-neuroradiological classification [44]. The other type of non-terminal myelocystocele is the complete form in which a hydromyelic cavity is continuous with the ependymal canal of the spinal cord (Fig. 7) [45]. While the brain can be normal in these entities, an association with obstructive hydrocephalus and hindbrain herniation has been described, though the incidence is difficult to determine given the relative rarity of this entity coupled with inconsistently used terminology in the literature.

### Lipomyelomeningocele

**Fig. 8:**
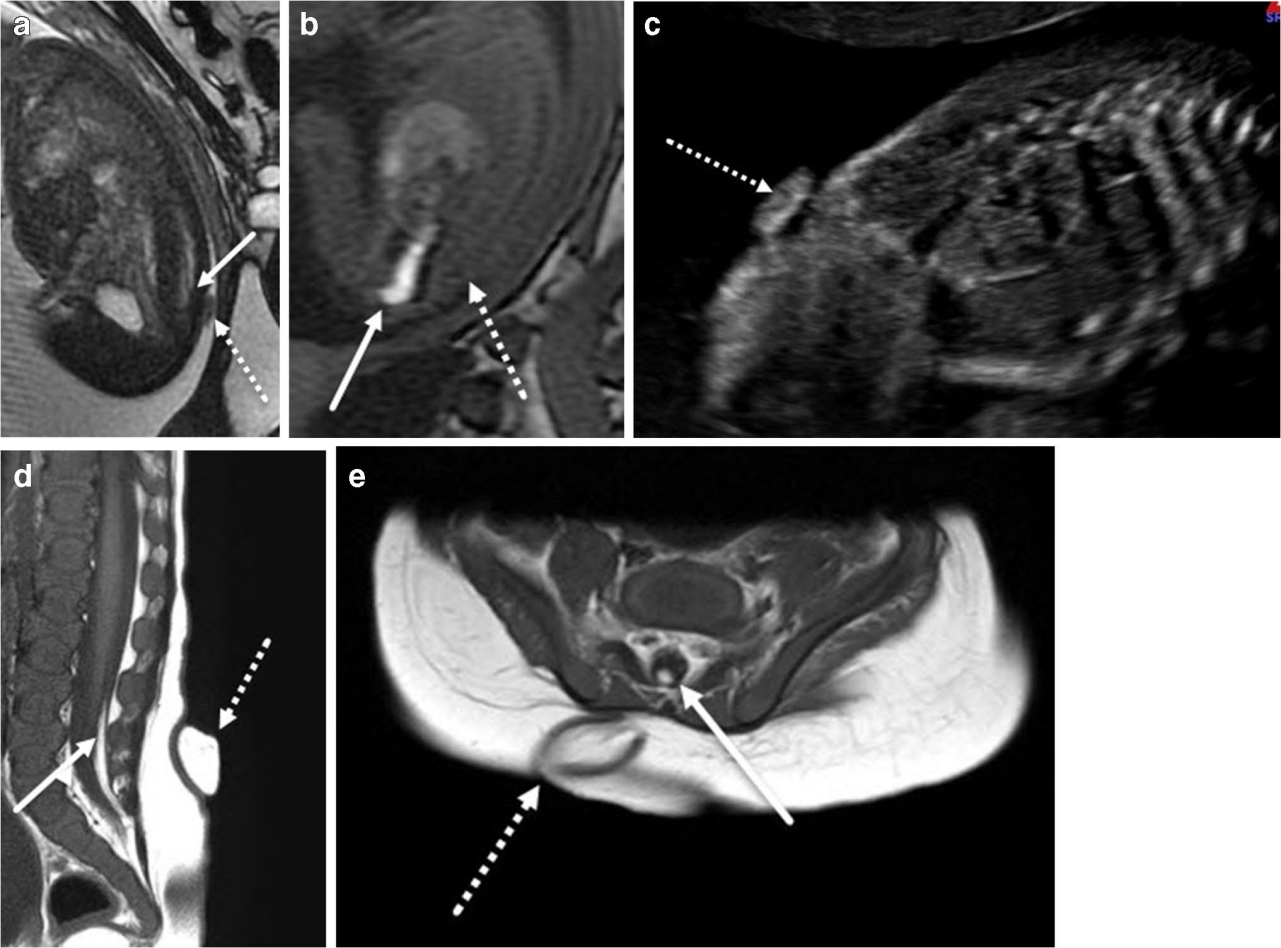
**a-f** Lipomyelocele with subcutaneous appendage. Sagittal BTFE/FIESTA image (**a**) in a 23-week 2-day gestational age fetus demonstrating low conus with neural placode at the caudal end of the thecal sac (*white arrow*) with a small overlying subcutaneous appendage (*white dashed arrow*). Sagittal T1-weighted image (**b**) demonstrates lack of appreciable T1 hyperintense fat associated with the spinal dysraphism (*white dashed arrow*) and normal rectal meconium signal (*white arrow*). Corresponding parasagittal fetal ultrasound image (**c**) shows the small subcutaneous appendage. Sagittal (**d**) and axial (**e**) T1-weighted images from postnatal MRI at 3 months of age demonstrate intradural lipoma in association with a low conus, the placode-lipoma interface within the spinal canal. Dorsal subcutaneous fatty appendage (*white dashed arrow*) is seen overlying the lumbosacral spine

Lipomyelomeningoceles and lipomyeloceles are both CSD characterized by an elongated spinal cord with a dorsal spinal dysraphic defect with lipomatous fatty tissue creating a placode-lipoma interface [4]. These are also sometimes referred to as “lipoma with dorsal defect” and are the most common CSD encountered in clinical practice [46]. When the placode-lipoma interface is located within the spinal canal, it is referred to as a lipomyelocele, and when located outside of the spinal canal, it is referred to as a lipomyelomeningocele, though these two entities do fall along a spectrum and can be difficult to distinguish from each other even postnatally. In addition, on prenatal imaging, the placode-lipoma interface is often not clearly delineated because of poor visualization of fatty tissue in the fetus due to underdevelopment of fat and the technical limitations of T1-weighted imaging in the fetus [47]. However, expansion of the meninges surrounding the neural placode is characteristic of a lipomyelomeningocele and should be a differential consideration when a skin-covered defect with a sac containing neural elements is identified. The brain is typically normal; however, a mildly increased incidence of cerebellar ectopia is seen in those with lipomyelomeningoceles compared with the general population [46]. Lipomyelomeningoceles and lipomyeloceles can also be seen in association with dorsal appendages or vestigial tails in the fetus, such that identification of a vestigial tail on fetal imaging warrants postnatal MRI and neurosurgical follow-up (Fig. 8) [48].

## Closed spinal dysraphism: complex, without a sac

### Neurenteric cyst

This CSD is characterized by a cyst along the neuroaxis that is lined with cuboidal or columnar epithelium. Neurenteric cysts are usually at least partially intraspinal, although they can be paraspinal and are often associated with segmentation anomalies of the adjacent vertebral bodies [49]. While ultimately a histologic diagnosis, this is a differential to consider when an intraspinal or paraspinal cyst is identified in the fetus. While these can be seen in isolation, they are associated with other syndromes including Klippel-Feil; vertebral defect, anal atresia, congenital heart defect, trachea-esophageal fistula, esophageal atresia, renal anomalies, limb anomalies (VACTERL) association; and OEIS. These are also commonly seen in association with other CSD including split cord malformation, intradural lipoma, and dorsal dermal sinus tract [50].

### Split cord malformation

**Fig. 9:**
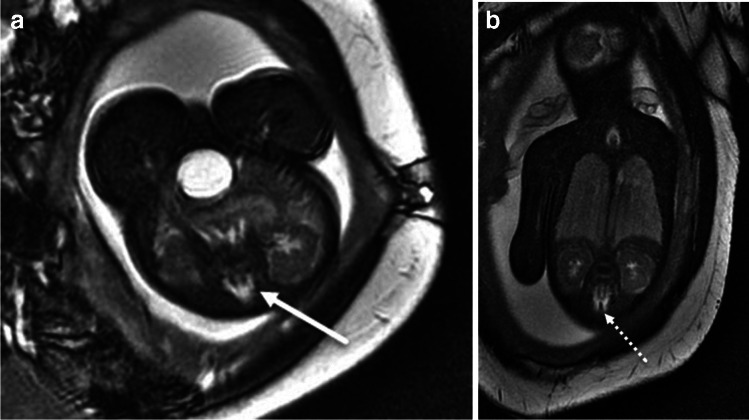
**a-b** Split cord malformation type I. Axial (**a**) and coronal (**b**) BTFE/FIESTA images in a 31-week 6-day gestational age fetus with split cord malformation (*solid arrow*) and an associated osseous cleft (*dashed arrow*)

This CSD is the sagittal division of the spinal cord into two hemicords [51]. The terms “diastematomyelia” and “diplomyelia” are also used to describe this entity, but these terms have fallen out of favor in the neurosurgical literature given the challenges in differentiating between the two on imaging and even in the operating room. There are two types of split cord malformation described, which have implications for surgical management. Type I split cord malformation consists of two separate thecal sacs, usually in association with a bony or cartilaginous spur (Fig. 9) [52]. Type II split cord malformation is contained within a single thecal sac, usually with a thin separating fibrous band (Fig. 3c-d). The hemicords can have variable symmetry and often unite below the cleft [53]. Type II can be seen in association with OSD, though fetal MRI is limited in its ability to identify this consistently [27]. Prophylactic surgery for untethering is generally recommended for split cord malformation, especially in type I, with resection of the spur and lysis of associated fibrous adhesions [54, 55].

### Caudal agenesis

**Fig. 10:**
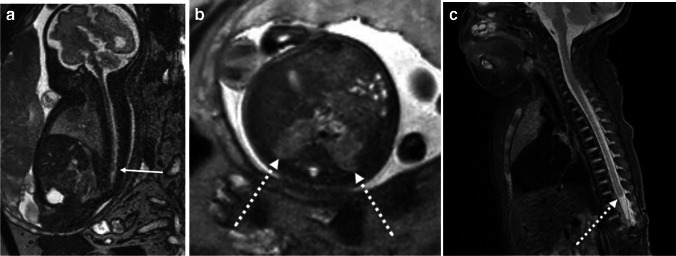
**a-c** Caudal agenesis group 1. Sagittal (**a**) BTFE/FIESTA and axial (**b**) T2-SSFSE images of a 29-week 2-day gestational age fetus with absence of the lumbosacral spine and a high blunted conus medullaris (**a**, *solid arrow*) and a horseshoe kidney (**b**, *dashed arrows*). Sagittal T2 FS image at 12 days of age again showing a high blunted conus (**c**, *dashed arrow*) and absent lumbosacral spine

**Fig. 11:**
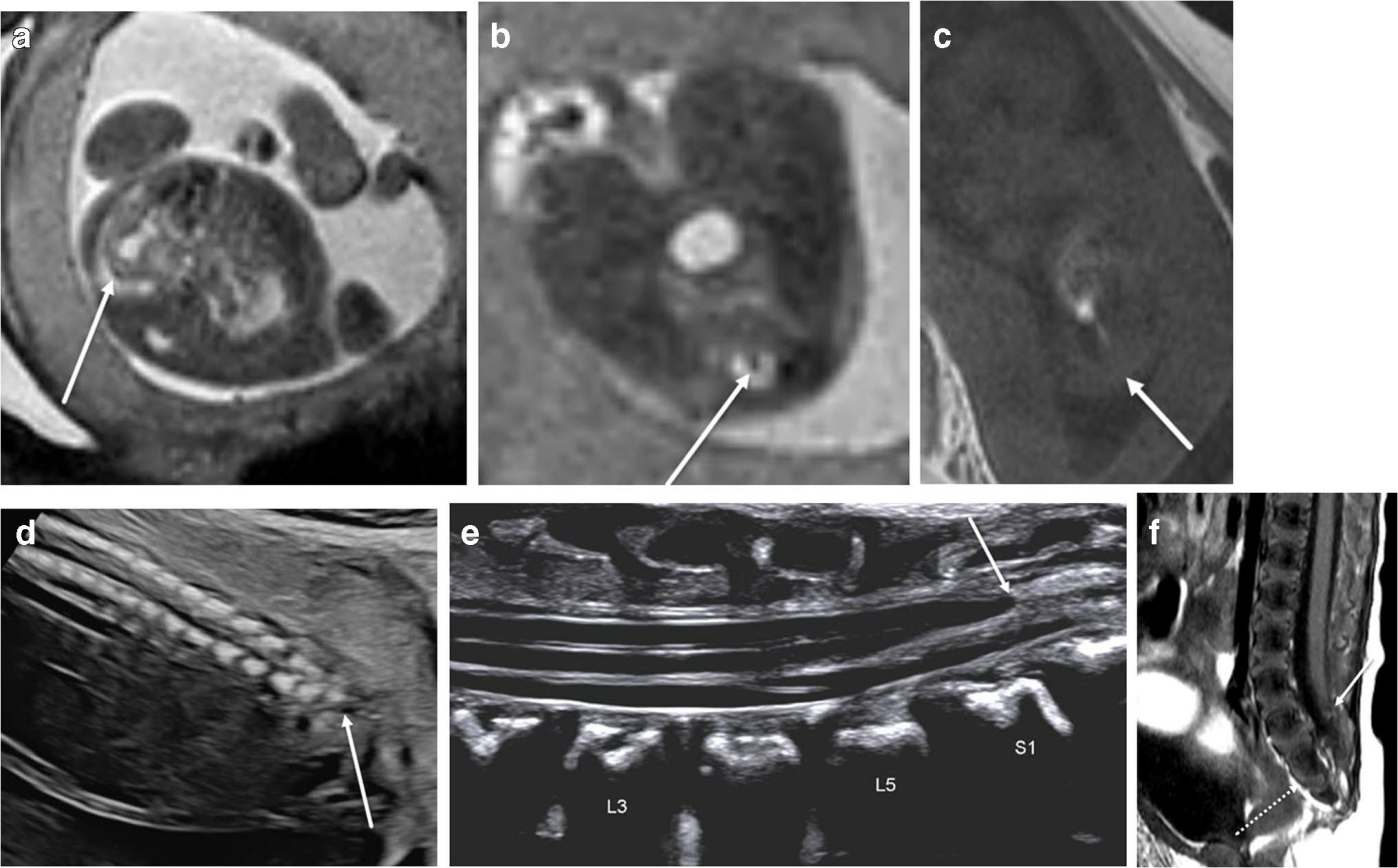
**a-f** Caudal agenesis group 2. Axial T2-SSFSE images through the abdomen (**a**) and pelvis (**b**) in a 20-week 6-day gestational age fetus demonstrate a solitary low-lying right kidney (**a**, *solid arrow*) and a low-lying conus medullaris (**b**, *solid arrow*). Sagittal T1-weighted image (**c**) demonstrates the absence of normal T1-hyperintense rectal meconium signal (*solid arrow*) consistent with anorectal malformation. Sagittal fetal ultrasound image (**d**) demonstrates sacral hypoplasia (*solid arrow*). Postnatal spine ultrasound at 1 day of age (**e**) confirms low-lying conus medullaris at the S1 level (*solid arrow*). Sagittal T1-weighted image at 5 days of age (**f**) again shows the low-lying conus (*solid arrow*) and sacral hypoplasia (*dashed arrow*)

**Fig. 12:**
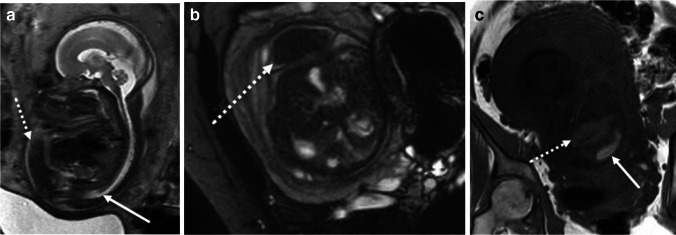
**a-c** Sirenomelia. Sagittal T2-SSFSE (**a**) and axial BTFE/FIESTA (**b**) images in a 26-week 1-day gestational age fetus demonstrate anhydramnios secondary to bilateral renal agenesis and a single thickened lower extremity (*dashed arrow*). There are a high blunted conus medullaris (**a**, *solid arrow*) and the absence of the lower lumbar and sacral spine. Sagittal T1-weighted image demonstrates dilated distal colon (*solid arrow*) just below the liver (*dashed arrow*) consistent with a high anorectal malformation

This spectrum of abnormalities, more commonly referred to as caudal regression, is primarily characterized by congenital absence of a portion of the caudal spine. Caudal agenesis is commonly associated with anorectal and genitourinary anomalies. It presents as a wide spectrum of aberrations in the fetus ranging from isolated coccygeal aplasia (which cannot be confidently diagnosed prenatally) to sirenomelia [56]. Caudal agenesis can be divided into two groups: Group 1 patients have a high (L1 or higher) blunted conus and tend to be associated with more severe osseous, anorectal, and genitourinary malformations (Fig. 10). Group 2 patients have a low-lying tethered cord and are often associated with additional CSD such as an intradural lipoma (Fig. 11) [57, 58]. Group 2 patients are typically candidates for neurosurgical intervention for tethered cord release, while group 1 patients generally do not have an identifiable tethering lesion amenable to surgical intervention. Sirenomelia (a.k.a. “mermaid syndrome”), a rare condition with partial absence of the caudal spine in association with fusion of the lower extremities, is believed by most authors to be on the severe end of the caudal agenesis spectrum, though some consider it to be a clinically and embryologically distinct entity [59]. Sirenomelia is typically associated with more severe gastrointestinal and genitourinary anomalies including anorectal atresia, renal agenesis, bladder agenesis, urethral agenesis, single umbilical artery, and ambiguous genitalia. Oligohydramnios and anhydramnios are also common in sirenomelia (Fig. 12) [60].

### Segmental spinal dysgenesis

**Fig. 13:**
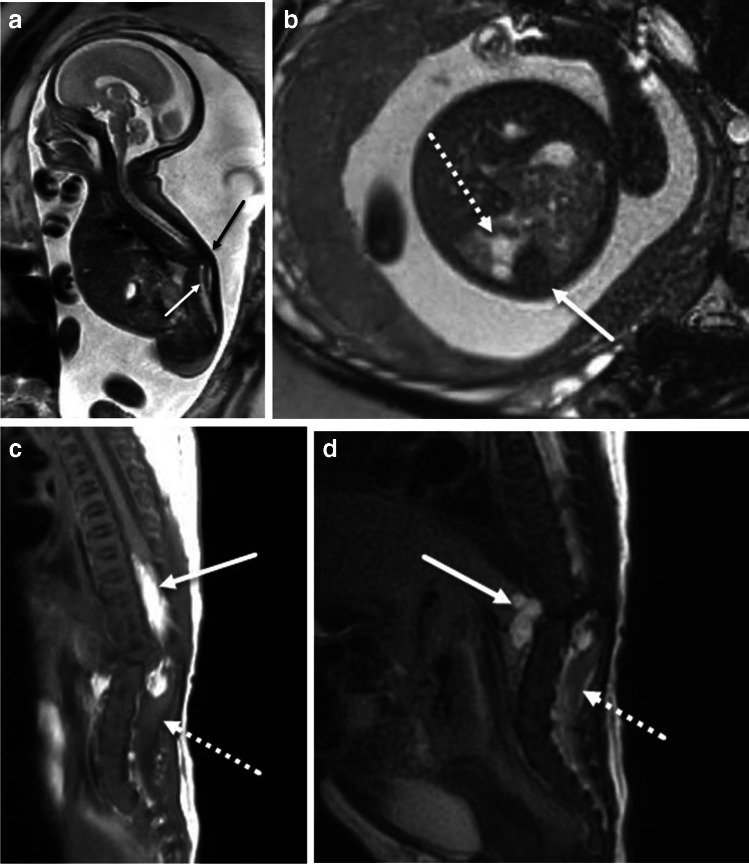
**a-d** Segmental spinal dysgenesis. Sagittal T2-SSFSE (**a**) and axial BTFE/FIESTA (**b**) images from fetal MRI at 26 weeks 1 day gestational age demonstrate focal kyphosis (*black arrow*) with effacement of the spinal canal (**b**, *white arrow*). There is characteristic thickening of the lower spinal cord with low-lying conus (**a**, *white arrow*). Note is also made of right paraspinal cysts associated with the spinal column abnormalities (**b**, *white dashed arrow*) suspicious for neurenteric cysts. Sagittal T1-weighted (**c**) and T2-FSE (**d**) images of the spine at birth again show thickened low-lying conus (*white dashed arrow*). There is also a large amount of intradural fat seen (**c**, *white arrow*), which was not seen on the previous fetal imaging. Suspected neurenteric cysts (**d**, *white arrow*) again demonstrated

This rare CSD is characterized by a focal deformity of the lumbar or thoracolumbar spine resulting in focal kyphosis or disarticulation of the spine. The spinal cord is either very thin or even absent at the level of the defect; however, it is normal or thickened caudal to the defect (Fig. 13) [61]. Clinically, these patients are usually paraplegic, though rare cases of only mild lower extremity motor impairment have been described. While this entity usually presents as a CSD, it can rarely present in the setting of OSD and Chiari II malformation [62].

## Closed spinal dysraphism: simple, without a sac

### Overview of entities

In the clinical-neuroradiological classification, there are a number of simple dysraphic states described, including, but not limited to, intradural lipoma, filum terminale lipoma, and tight filum terminale. These entities are very challenging to confidently diagnose on fetal imaging in isolation. Dorsal dermal sinus tract is another CSD described in the clinical-neuroradiological classification under “complex dysraphic state without a subcutaneous mass.” In isolation, this is also very challenging, if not impossible, to identify on fetal imaging. Normal variants such as a persistent terminal ventricle or filar cysts also, in the authors’ opinion, cannot be confidently identified on fetal imaging. When approaching spinal dysraphism in the fetus, it is important to keep these entities in mind in order to understand our limitations in fetal imaging.

### Low conus

Positioning of the conus medullaris provides the highest diagnostic accuracy in identifying tethered cord syndrome, which is a clinical diagnosis [63]. However, confident identification of the conus medullaris position can be challenging, especially early gestation, not only due to the limitations of accurate vertebral body assignment on fetal imaging but also because of the relative lack of normal fetal imaging data. Most pediatric neurosurgeons accept the lower aspect of L2 as the lower limit of normal in the postnatal period for conus tip position; however, level cutoffs in the prenatal period are less clear. There are multiple papers describing conus ascent in the spinal canal in the developing fetus and early infancy [64–66]. Though the spinal cord can be challenging to evaluate consistently on fetal ultrasound, one paper looking at fetal ultrasound data described a conus tip position as low as the L4 level before 24 weeks gestational age as within the range of normal [67]. In a smaller study examining ex-vivo postmortem fetal MRI, it was observed that the conus lay at the L2-L5 levels before 35 weeks and at the L1-L3 levels after 35 weeks gestational age [68]. The fetal kidneys can also potentially be used as a screening landmark, such that if the conus position is below the lower pole of the kidneys at any gestational age, the possibility of low conus should be raised [69]. Low conus can be seen in isolation or in association with other CSD. When identified on fetal imaging, this finding warrants postnatal ultrasound or MRI to confirm or refute low conus positioning.

## Conclusion

In this review, we described a method of approaching spinal dysraphism on fetal imaging, based on the previously published clinical-neuroradiological classification. We reviewed the most common congenital spinal anomalies seen in our fetal imaging practice and provided multiple case examples in a pictorial review. This approach can be used in the prenatal diagnosis and classification of fetal spinal dysraphism in routine clinical practice.

## Supplementary Information

Below is the link to the electronic supplementary material.

Supplementary file1 (DOCX 1079 KB)

Supplementary file2 (MP4 1977 KB)

Supplementary file3 (MP4 690 KB)

Supplementary file4 (MP4 994 KB)

Supplementary file5 (MP4 385 KB)

Supplementary file6 (MP4 1450 KB)

Supplementary file7 (MP4 1350 KB)

Supplementary file8 (MP4 566 KB)

Supplementary file9 (MP4 1343 KB)

Supplementary file10 (MP4 687 KB)

Supplementary file11 (MP4 849 KB)

## Data Availability

No datasets were generated or analysed during the current study.
