# Supplementary material for: Fetal imaging approach to spinal dysraphism diagnosis
Source: Pediatr Radiol. 2025 Jun 20;56(8):1664–77. doi: 10.1007/s00247-025-06287-2 (PMC13407996; doi:10.1007/s00247-025-06287-2)
Supplement: Supplementary file 1 — Supplementary file1 (DOCX 1079 KB) [file 247_2025_6287_MOESM1_ESM.docx]

**Supplementary Materials**

| **Sequence** | **FOV** | **Imaging Plane (s)** | **Slice Thickness** | **Minimum Number of Times** |
| --- | --- | --- | --- | --- |
| FIESTA/TrueFISP/BTFE | Maternal Uterus | Axial, Coronal, Sagittal | 5 mm | 1 |
| HASTE/SS-FSE/SS-TSE | Brain | Axial, Coronal, Sagittal: attention midline anatomy | < 24 weeks 3 mm  ≥ 24 weeks 4 mm | 2 |
| FIESTA/TrueFISP/BTFE | Brain | Axial, Coronal, Sagittal | 4 mm | 1 |
| DWI | Brain | Axial | 4 mm | 1 |
| EPI/MPGR (Blood) | Brain | Axial | 3 mm | 1 |
| DTI 15 Directions | Brain | Axial | 4 mm | 1 |
| HASTE/SS-FSE/SS-TSE | Spine | Axial, Coronal, Sagittal | < 24 weeks 3 mm  ≥ 24 weeks 4 mm | 1 |
| FIESTA/TrueFISP/BTFE | Spine | Axial, Coronal, Sagittal | 4 mm | 2 axial and sagittal, 1 coronal |
| T1 SPGR | Body | Coronal and Sagittal | 5 mm | 1 |
| EPI/MPGR (Black Bone) | Spine | Sagittal and Coronal | 5 mm | 1 |

**Supplementary Table S1:** Fetal MRI Spinal Dysraphism Protocol used at Cincinnati Children’s Hospital Medical Center, last updated in 2024. All sequences are repeated as requested by the radiologist. FOV: field of view, FIESTA: fast imaging employing steady-state acquisition, TrueFISP: fast imaging with stead-state free precession, BTFE: balanced turbo field echo, HASTE: half-fourier acquisition single-shot turbo spin-echo, SS-FSE: single shot fast spine echo, SS-TSE: single shot turbo spin echo, DTI: diffusion tensor imaging, DWI: diffusion weighted imaging, EPI: echo planar imaging, MPGR: multiplanar gradient-recalled, SPGR: spoiled gradient echo recalled


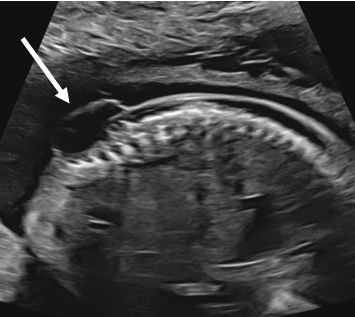


**Figure S2c:** Sagittal US image of the spine in same patient as a-b demonstrating the lumbosacral myelomeningocele (arrow). **See Video S2d** for more detail.


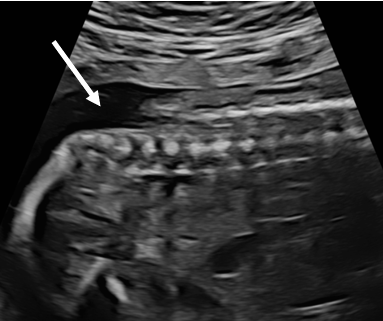


**Figure S3c:** Sagittal fetal US image of the spine in same patient as c-d demonstrating the lumbosacral myelocele (arrow) with no visible sac. **See Video S3d** for more detail.


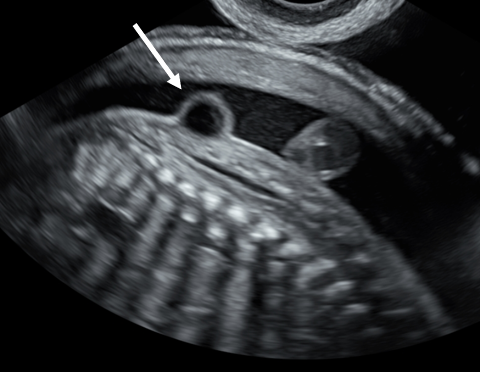


**Figure S4d:** Sagittal fetal US image of the spine in the same fetus as a-b demonstrating the sacral meningocele (arrow) with thick sac wall in continuity with the adjacent subcutaneous tissues. **See Video S4e** for more detail.


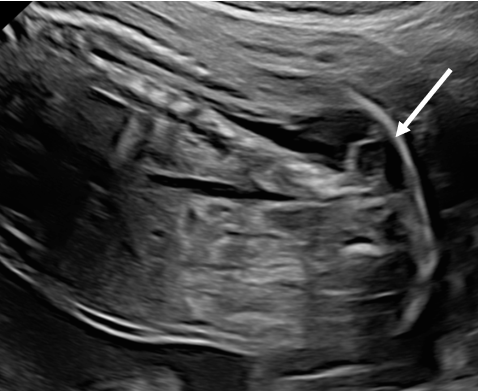


**Figure S5d:** Sagittal fetal US image of the spine in the same fetus with terminal myelocystocele (arrow) demonstrating the “cyst within a cyst” appearance of the sac. **See Video S5e** for more detail.


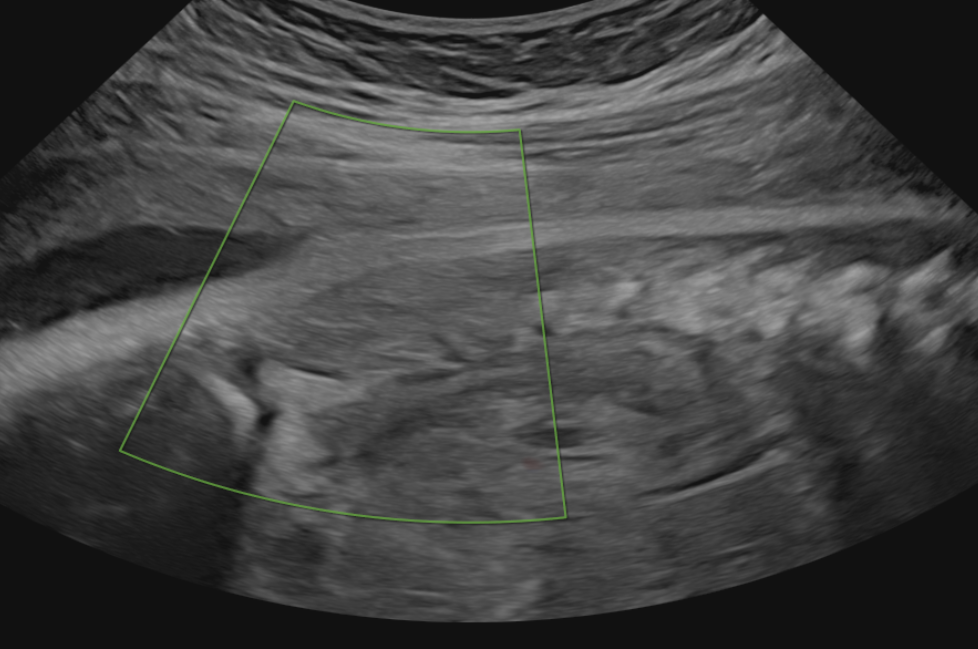


**Figure S9c:** Sagittal fetal US image of the spine in the same fetus with color doppler interrogation over an area of thickening of the subcutaneous tissues over the lumbosacral spine. **See Video S9d** for more detail.


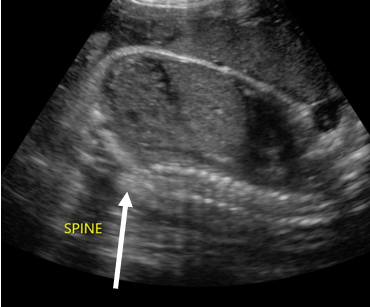


**Figure S10d:** Sagittal fetal US image of the spine in the same fetus demonstrating absence of the lower lumbar and sacral spine (arrow). **See Video S10e** for more detail.


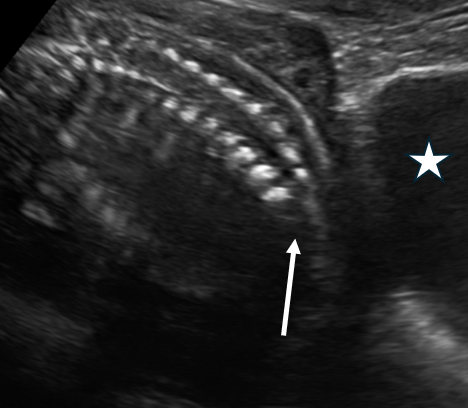


**Figure S12d:** Sagittal fetal US image of the spine in the same fetus with sirenomelia demonstrating absence of the lower lumbar and sacral spine (arrow). The maternal bladder (star) is in the field of view. **See Video S12e** for more detail.


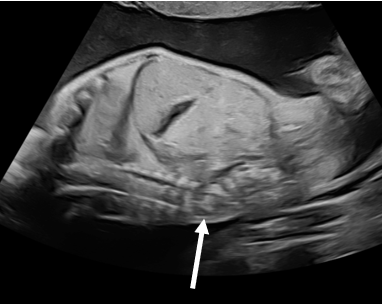


**Figure S13e:** Sagittal fetal US image of the spine in the same fetus with segmental spinal dysgenesis demonstrating focal kyphosis of the midthoracic spine (arrow). See **Video S13f** for more detail.
